# Increased Use of Antibiotics in the Intensive Care Unit During Coronavirus Disease (COVID-19) Pandemic in a Brazilian Hospital

**DOI:** 10.3389/fphar.2021.778386

**Published:** 2021-12-10

**Authors:** Alice Ramos Oliveira Silva, Diamantino Ribeiro Salgado, Luis Phillipe Nagem Lopes, Débora Castanheira, Isabel Cristina Martins Emmerick, Elisangela Costa Lima

**Affiliations:** ^1^ Pharmacy School, Federal University of Rio de Janeiro, Rio de Janeiro, Brazil; ^2^ Clementino Fraga Filho University Hospital, Federal University of Rio de Janeiro, Rio de Janeiro, Brazil; ^3^ National School of Public Health Sergio Arouca, Rio de Janeiro, Brazil; ^4^ Division of Thoracic Surgery, Department of Surgery, UMass Chan Medical School, Worcester, MA, United States

**Keywords:** anti-infective agents, COVID-19, intensive care units, bacterial infection, drug resistance, bacterial, coinfection

## Abstract

**Background:** Microbial drug resistance is one of the biggest public health problems. Antibiotic consumption is an essential factor for the emergence and spread of multiresistant bacteria. Therefore, we aimed to analyze the antibiotics consumption in the Intensive Care Unit (ICU), identifying trends in the antibiotics use profile and microbiological isolates throughout the COVID-19 pandemic.

**Methods:** We performed this retrospective observational study in intensive care units of a Brazilian tertiary hospital from January 2019 to December 2020. The primary outcome was antimicrobial consumption in the ICU, measured by defined daily doses (DDDs) per 100 bed-days. As a secondary outcome, bacterial infections (microbiological isolates) were calculated in the same fashion. Outcomes trends were analyzed using Joinpoint regression models, considering constant variance (homoscedasticity) and first-order autocorrelation assumptions. A monthly percent change (MPC) was estimated for each analyzed segment.

**Results:** Seven thousand and nine hundred fifty-three patients had data available on prescribed and received medications and were included in the analyses. Overall, the use of antibiotics increased over time in the ICU. The reserve group (World Health Organization Classification) had an increasing trend (MPC = 7.24) from February to April 2020. The azithromycin consumption (J01FA) increased rapidly, with a MPC of 5.21 from January to April 2020. Polymyxin B showed a relevant increase from March to June 2020 (MPC = 6.93). The peak of the antibiotic consumption of Reserve group did not overlap with the peak of the pathogenic agents they are intended to treat.

**Conclusion:** Overall antimicrobial consumption in ICU has increased in the context of the COVID-19 pandemic. The peaks in the antimicrobial’s use were not associated with the rise of the pathogenic agents they intended to treat, indicating an empirical use, which is especially concerning in the context of treating multidrug-resistant (MDR) infections. This fact may contribute to the depletion of the therapeutic arsenal for MDR treatment.

## Introduction

Antibiotic resistance is a significant public health problem and one of the greatest challenges of our time (Centers for Disease Control and Prevention, 2021a). A contributing factor to antibiotic resistance is the misuse of antibiotics in hospitals (Food and Drug Administration, 2020), an estimated 25–50% of antimicrobials prescribed in hospitals are unnecessary or inappropriate, directly impacting antimicrobial resistance (Da Silva et al., 2021). Furthermore, the ability of microorganisms to become resistant to previously susceptible drugs is superior to the capacity of the pharmaceutical industry to introduce new antimicrobials onto the market (Fair and Tor, 2014).

Although clinical experience with previous viral epidemics suggests risks of bacterial co-infection (Chung and Huh, 2015), since the beginning of the COVID-19 pandemic experts have been warning about the risks of antibiotic overuse (Centers for Disease Control and Prevention, 2021a; Rodríguez-Baño et al., 2021). In the hospital’s intensive care units (ICU), the severity of the diseases treated and the multiple interventions to the patients are expected a high antimicrobial use (Da Silva et al., 2021). Therefore antimicrobial monitoring use in the case of the pandemic is crucial to identify concerning signs of misuse or overuse. Studies conducted in several countries found an increase in antimicrobial consumption in ICUs during the pandemic (Guisado-Gil et al., 2020; Rawson et al., 2020; Grau et al., 2021b; Ng et al., 2021).

Many low and middle-income countries, Brazil among them, have struggled to implement a robust antimicrobial management program. For instance, more than half of Brazilian hospitals do not have an antimicrobial stewardship program (ASP) (Agência Nacional de Vigilância Sanitária, 2020). These fragmented and deficient ASP initiatives deteriorated further during the ongoing COVID-19 pandemic (Ul Mustafa et al., 2021). Furthermore, the lack of complete public data from the Brazilian Federal Agencies during the pandemic makes this investigation challenging in Brazil (Ministry of Health/National Health Surveillance Agency/Collegiate Board) compared to the availability of information in other countries such as those Centers for Disease Control and Prevention (CDC) (Centers for Disease Control and Prevention, 2021b).

Albeit Brazil was one of the world epicenters of COVID-19 (Hallal and Victora, 2021), national, regional data on antimicrobial consumption in hospitals during the SARS-COV-2 pandemic is unknown. Therefore is fundamental to increase the knowledge of antimicrobial consumption and its relevance for antimicrobial resistance.

This study aimed to analyze the antibiotics consumption in intensive care units (ICU) of a private hospital in Rio de Janeiro, Brazil, from January-2019 to December-2020, identifying the trends of antibiotics use profile during the COVID-19 pandemic.

## Material and Methods

### Study Design and Setting

We conducted the study at a medium-sized tertiary hospital in Rio de Janeiro, Brazil, with 172 beds, 52 of which are ICU. It was one of the state’s reference hospitals to care for critically ill patients affected by COVID-19. The average occupancy rate of beds in the five intensive care units studied during the period was around 98%.

### Data Sources and Collection

We included patients admitted to the ICU for at least 24 h from January 2019 to December 2020. We collected individual level data retrospectively from a central pharmacy distribution system. Thus physicians were not aware of the study when prescribing the anti-infectives, reducing the likelihood of bias. We characterized the study population (clinical and demographic data) upon admission to the ICU.

Our primary outcome of interest was trending on antibiotic consumption. We expressed the volume of antimicrobials as monthly Defined Daily Dose per 100 beds-days (DDD/beds-day), as established by the WHO (World Health Organization, 2020). DDD is defined as the presumed average maintenance dose per day for a drug used for its primary indication in adults. The World Health Organization (WHO) Collaborating Center attributes the value of DDD using established principles (Hutchinson et al., 2004).

The antimicrobials were classified using their Anatomical Therapeutic Chemical Classification System (ATC) code (Hutchinson et al., 2004) and WHO AWaRe Classification Database of Antibiotics for evaluation and monitoring of use (World Health Organization, 2019a).

Our secondary outcome was microbiological data aiming to describe the trends on microbiota type of infection during the period analyzed. Considering the patients admitted to the ICU, we obtained the microbiological data, including all cultures with microbiological growth. This data was available in the institution’s clinical analysis service database, given that diagnostic culture collection was a practice from the investigated hospital (Kalil et al., 2016; Torres et al., 2017; Levy et al., 2018).

We analyzed all cultures with microbiological growth collected from patients, except for screening cultures. We grouped the microorganisms into non-fermenting Gram-negative bacilli (NFGNB), Gram-negative fermenting bacilli (GNBF), Gram-positive cocci (GP), and fungi. We summarized the number of microbiological isolates per month divided by beds-day and multiplied by 100. Multidrug-resistant (MDR) was defined as a microorganism resistant to at least one agent in three categories (Magiorakos et al., 2012).

### Statistical Analysis

We performed descriptive analysis, including univariate and bivariate analysis, to characterize the study population and the antibiotic consumption and to identify differences in the study’s years. We used frequency, medians, interquartile intervals, and proportions and performed chi-square and Mann-Whitney to identify differences statistically significant.

The data were analyzed under the assumption of constant variance (homoscedasticity) and first-order autocorrelation. In addition, a monthly percent change (MPC) in DDD for each line segment was estimated. The rates are assumed to change at a constant percentage monthly. The MPC was tested to determine whether a difference exists from this null hypothesis (Kim et al., 2000). We analyzed data by ATC, AWaRe classification, and drug level.

In this analysis, we made the option of using joinpoint regression analysis instead of interrupted time series (ITS). We investigated the possibility of implementing ITS using ARIMA and PRAIS models to analyze the data. Nevertheless, the Durbin-Watson was indicating an important autocorrelation of the residues and therefore ITS was not appropriate.

In summary, the antibiotic use series is non-stationary and suffers multiple stochastic shocks during the analyzed period. Considering that these stochastic shocks are not exclusive uni-root, seasonal, or time dependent, it is not possible to perform its transformation in a stationary series without butchering its main characteristics. Therefore, the series is not a candidate to ARIMA in the Box-Jenkins model (George E. P. Box et al., 1976).

ITS is a very valuable technique, and it might be possible to be used in the future for antibiotic analysis. For this study, we evaluate that the COVID-19 was a very dynamic scenario, and for antibiotic use, there were many time-varying confounders that would be very difficult to control for, such as prescription patterns, fake news about the efficacy of some medicines, among others.

Joinpoint regression is a data-driven analysis and, therefore, more appropriate for this scenario since it would be challenging to determine the exact beginning of the pandemic. In this sense, the trend analysis allows one to investigate the pattern of antibiotic consumption alongside the pandemic patterns, identifying the waves in specific antibiotics utilization.

In the final model, each joinpoint informs a statistically significant change in trends (increase or decrease), and each of those trends is described by an MPC (Kim et al., 2000, 2000; Qiu et al., 2009). The analyses were conducted using Joinpoint Regression Program version 4.9.0.0, a trend analysis software developed by the US National Cancer Institute (US NCI) to analyze data from the Surveillance Epidemiology and End Results Program (SEER) (National Cancer Institute, 2020).

The Ethics and Local Research Committee approved the study under CAAE: 25683019.4.0000.5249.

## Results

The study included 7,953 patients admitted to the ICU of a Brazilian tertiary hospital from January 2019 to December 2020. The number of hospitalized patients decreased, but their severity increased in 2020 (the year the COVID-19 pandemic started) compared to 2019, before the pandemic (Table 1). The median consumption of all antimicrobials analyzed was higher in 2020 compared to 2019 (127.4 DDD/beds-day versus 115 DDD/beds-day) (*p* = 0.068). An increase in the consumption of antibiotics classified as Reserve was also observed: the DDD/100 beds days of polymyxin B and daptomycin and linezolid changed from 6.7, 0.0, and 0.68 to 16.8, 0.7, and 1.5, respectively. (*p* = 0.005, 0.043 and 0.020) (Table 2).

**TABLE 1:** Characterization of the study population (Rio de Janeiro, Brazil).

Variables	Total N = 7,953	Year		
		2019	2020	P-valor
		N = 4,299	N = 3,654	
Demographic characteristics				
Female sex	3,915 (49.2)	2,209 (51.4)	1,706 (46.7)	**<0.001**
Age	68 (51–80)	68 (51–80)	67 (51–80)	0.165
BMI	27 (24–30)	27 (24–30)	27 (24–30)	0.060
Clinical characteristics				
Charlson CCI				
Low (0 point)	3,296 (41.4)	1,819 (42.3)	1,477 (40.4)	0.093
Medium (1–2 points)	2,872 (36.1)	1,553 (36.1)	1,319 (36.1)	0.702
High (3–4 points)	729 (9.2)	418 (9.7)	311 (8.5)	0.066
Very high (≥5 points)	1,056 (13.3)	509 (11.8)	547 (15.0)	**<0.001**
Palliative care	128 (1.6)	76 (1.8)	52 (1.4)	0.259
Septic shock	2,140 (26.9)	899 (20.9)	1,241 (34.0)	**<0.001**
Mechanical ventilation	829 (10.4)	315 (7.3)	514 (14.1)	<**0.001**
Infection/Sepsis	2,492 (31.1)	1,147 (26.7)	1,345 (36.8)	**<0.001**
SAPS 3	44 (36–52)	43 (34–51)	44 (37–53)	**<0.001**
ICU length of stay	2 (1–4)	2 (1–4)	2 (1–5)	**<0.001**
Hospital length of stay	6 (3–14)	5 (3–12)	7 (3–17)	**<0.001**

The categorical variables were expressed in absolute frequencies and, in between parenthesis, the relative frequencies. The continuous variables were expressed in median and, in between parenthesis, the 25–75% interquartile range. BMI, body mass index, Charlson ICC, charlson comorbidities index; SAPS, 3 = Simplified Acute Physiology Score 3, ICU, intensive care unit.

a Median and standard derivation. The *p*-value was obtained using the chi-square test for categorical variables and the Mann-Whitney test for continuous variables.

*p*-values in bold are statistically significant.

**TABLE 2:** Median, interquartile range of antimicrobial consumption in DDD from January 2019 to December 2020 (Rio de Janeiro, Brazil).

Antibiotics	Total	2019	2020	*p*-value
	Median (IQR)	Median (IQR)	Median (IQR)	
All antibiotics (J01 and J02)*	115.4 (107.8–129.4)	110.8 (105.6–117.9)	123.2 (113.8–145.4)	0.068
**Reserve**				
Ceftazidime/avibactam	1.3 (0.0–2.2)	1.3 (0.0–2.3)	1.3 (0.5–2.2)	0.919
Ceftozolone/tazobactam	0.6 (0.0–0.75)	0.5 (0.0–0.7)	0.7 (0.1–0.9)	0.313
Polymyxin B	12.9 (6.5–16.3)	6.7 (5.6–12.1)	16.8 (13.4–20.7)	**0.005**
Polymyxin E	0.0 (0.0–0.7)	0.0 (0.0–0.26)	0.68 (0.0–1.5)	**0.043**
Daptomycin	0.0 (0.0–0.7)	0.0 (0.0–0.0)	0.7 (0.0–1.1)	**0.020**
Linezolid	0.9 (0.6–1.8)	0.68 (0.1–1.25)	1.5 (0.8–2.0)	0.113
Tigecycline	7.5 (5.1–9.7)	9.3 (7.8–10.1)	5.6 (4.2–7.2)	0.089
**Watch**				
Azithromycin	8.7 (7.8–9.8)	8.1 (7.3–9.5)	9.7 (8.5–15.15)	0.057
Cefepime	0.5 (0.0–1.32)	1.3 (0.6–1.7)	0.2 (0.0–0.6)	0.074
Ceftazidime	2.7 (1.5–3.0)	2.8 (1.5–2.9)	2.5 (1.9–3.5)	0.713
Ceftriaxone	1.6 (0.9–2.5)	1.9 (1.2–2.8)	1.1 (0.8–1.8)	0.083
Cefuroxime	0.3 (0.0–0.7)	0.1 (0.0–0.49)	0.5 (0.2–0.8)	0.184
Ciprofloxacin	2.5 (1.4–3.9)	3.9 (2.6–5.2)	1.4 (1.1–2.5)	**0.001**
Ertapenem	1.0 (0.4–1.6)	1.3 (0.7–2.1)	0.5 (0.3–1.2)	0.060
Levofloxacin	1.2 (0.7–2.3)	0.9 (0.3–1.7)	1.5 (0.8–2.68)	0.242
Meropenem	18.3 (16.0–19.4)	19.9 (17.6–27.4)	16.7 (14.0–19.5)	0.128
Moxifloxacin	0.2 (0.0–0.5)	0.2 (0.1–0.2)	0.0 (0.0–0.3)	0.239
Piperacilin/tazobactam	14.3 (13.3–15.7)	13.9 (13.1–15.3)	14.6 (14.2–17.0)	0.267
Teicoplanin	6.4 (5.0–7.8)	5.8 (4.7–7.0)	6.9 (5.7–8.2)	0.319
Vancomycin	2.1 (1.2–3.3)	2.6 (1.4–3.5)	1.9 (1.2–2.9)	0.487
**Access**				
Ampicilin	1.7 (1.3–2.6)	1.7 (1.6–2.1)	1.9 (0.9–3.5)	0.843
Ampicilin/subactam	0.2 (0.0–0.6)	0.2 (0.0–0.7)	2.5 (0.1–0.6)	0.886
Amikacin	9.3 (6.5–12.4)	6.5 (5.2–9.1)	12.9 (10.8–17.4)	**0.002**
Amoxicillin and clavulanate	7.1 (6.0–8.0)	6.7 (6.2–7.7)	7.2 (2.6–9.8)	1.000
Metronidazole	2.6 (1.5–3.4)	3.2 (2.4–3.8)	2.0 (1.1–2.6)	**0.017**
**Not classified– antifungal**			** **	** **
Amphotercin	0.0 (0.0–0.9)	0.0 (0.0–0.0)	0.7 (0.1–1.5)	**<0.001**
Anidulafungin	3.1 (2.3–4.7)	3.1 (1.9–3.9)	3.1 (2.7–5.0)	0.511
Fluconazole	1.2 (0.8–1.4)	1.2 (0.3–2.2)	1.3 (0.8–1.8)	0.630
Voriconazole	0.4 (0.0–0.8)	0.6 (0.0–1.1)	0.1 (0.0–1.8)	1.000

Legend: IQR, interquartil rate; *ATC, classification.

*p*-values in bold are statistically significant.

**FIGURE 1:**
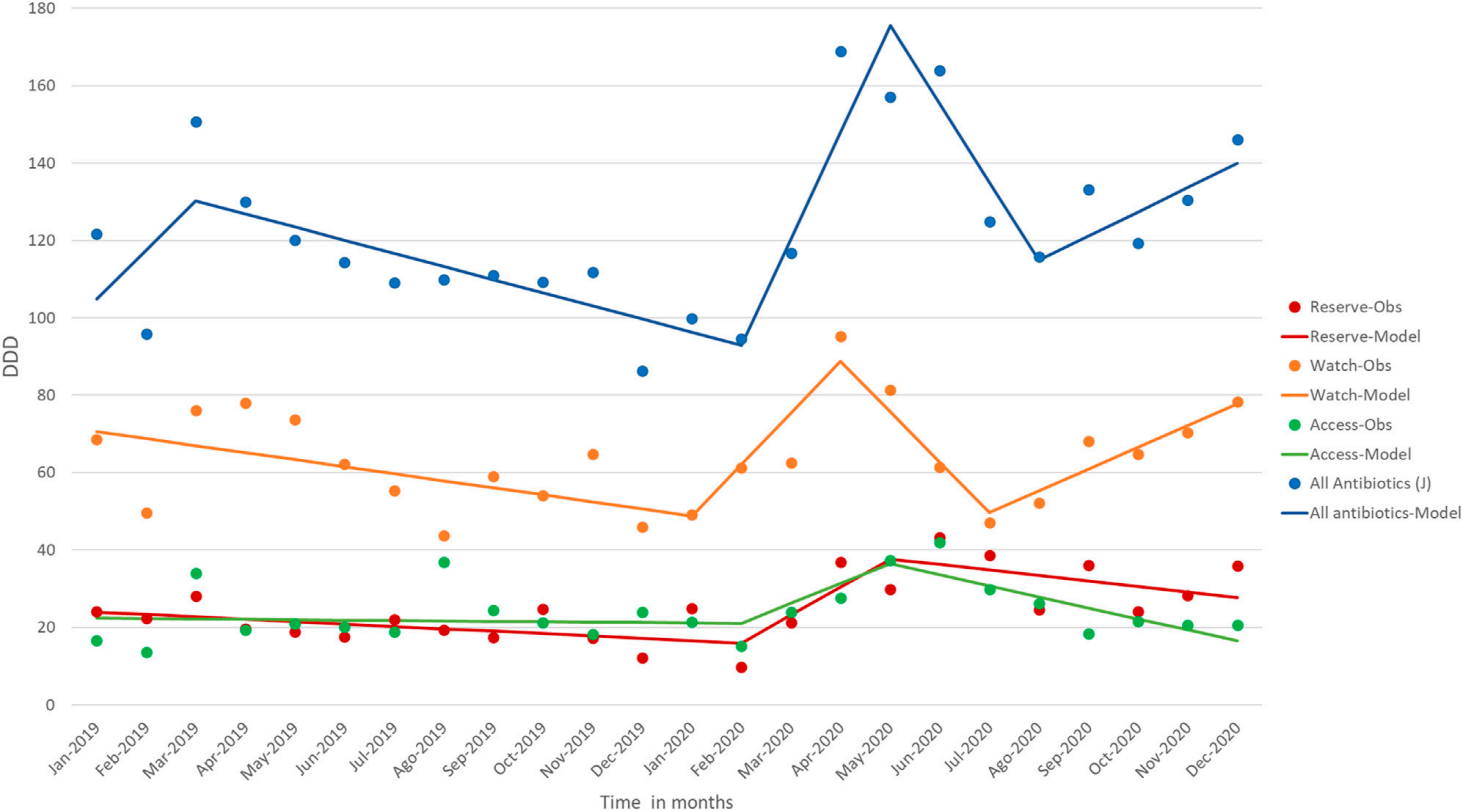
Antibiotic consumption (DDD) for Overall (J (J01 and J02)) and Access, Watch and Reserve from January 2019 to December 2020 (Rio de Janeiro, Brazil).

The antibiotics use increased over time in the ICU during the analyzed period (Figure 1). However, the microbiological isolates from ICU patients varied throughout 2019 and reduced in 2020. Microbiological isolates from ICU patients increased until April 2019 (January 2019 to April 2019, MPC = 6.02^∗^). From April 2019 to July 2019, there was a downward trend (MPC = - 3.76). From July 2019 to November 2019, there was an increase in isolates (MPC = 1.91^∗^). In November 2019, we observed a downward trend that continued until February 2020. From February 2020 to May 2020, there was an increase in isolates. Then, a downward trend continued until the end of the period analyzed (May/20-Dec/20 = MPC −0,49^∗^).

We observed an upward trend in antibiotic consumption for all AWaRe group classification. The Reserve group had an increasing trend from February to April 2020 (Monthly Percent Change MPC = 7.24), followed by a decrease until December 2020 (MPC = 1.42), placing it at a higher baseline when compared to the beginning of the period analyzed (Table 3 and Figure 1).

**TABLE 3:** Analysis of the consumption of antimicrobials for systemic use from January 2019 and 2020 by Joinpoint regression (Rio de Janeiro, Brazil).

Drug	Baseline		Trend 1		Trend 2		Trend 3		Trend 4	
	Period	MPC	Period	MPC	Period	MPC	Period	MPC	Period	MPC
AWARe Classification										
Access	Jan/19-Feb/20	−0.10	Feb-May/20	5.15	May-Dec/20	*−2.86				
Watch	Jan/19-Jan/20	*−1.82	Jan-Apr/20	13.30	Apr-Jul/20	−13.02	Jul-Dec/20	5.64		
Reserve	Jan/19-Feb/20	*0.61	Feb-May/20	7.24	May-Dec/20	1.42				
ATC Classification										
J (J01 and J02)	Jan-Mar/19	12.71	Mar/19-Feb/20	−3.39*	Feb-May/20	27.51	May-Aug/20	−20.19	Aug-Dec/20	6.28
J01AA (Tigecycline)	Jan/19-Dec/20	*−0.16								
J01CA (Ampicillin)	Jan/19-Dec/20	−0.05								
J01CR	Jan/19-Jan/20	−0.42	Jan-Apr/20	4.07	Apr-Dec/20	*−2.06				
Amoxicillin and clavulanate	Jan/19-May/20	*0.40	May-Dec/20	*1.82						
Ampicillin and sulbactam	Jan-Nov/19	*−0.13	Nov/19-Dec/20	*0.07						
Piperacillin and tazobactam	Jan-Set/19	0.46	Sep-Dec/19	−3.42	Dec/19-Mar/20	3.15	Mar-Dec/20	−0.08		
J01DC (Cefuroxime)	Jan/19- May/20	0.00	May-Dec/20	*1.92						
J01DD	Jan/19- Dec/20	−0.46								
Ceftazidime and avibactam	Jan-Jul/19	*0.71	Jul-Dec/19	−0.77	Dec/19- Dec/20	*0.25				
Ceftriaxone	Jan/19- Dec/20	−0.04								
Ceftazidime	Jan/19- Dec/20	0.00								
J01DE (Cefepime)	Jan-Apr/19	−0.54	Apr-Jul/19	1.55	Jul/19- Dec/20	*−0.14				
J01DH	Jan-Dec/19	*−0.49								
Ertapenem	Jan-Aug/19	*−0.38	Aug/19- Jan/20	0.27	Jan-May/20	-0.46	May- Aug/20	0.48	Aug- Dec/20	-0.27
Meropenem	Jan/19- Dec/20	*−0.41								
J01DI (Ceftozolone and tazobactam)	Jan/19- Dec/20	0.03								
J01MA	Jan/19- Jan/20	*−0.38	Jan-Dec/20	*0.26						
Levofloxacin	Jan/19- Dec/20	*0.09								
Moxifloxacin	Jan/19- Dec/20	*−0.01								
Ciprofloxacin	Jan/19- Dec/20	*−0.18								
J01FA (Azithromycin)	Jan/19- Jan/20	0.03	Jan-Apr/20	*5.21	Apr-Jul/20	*−6.13	Jul-Dec/20	*0.94		
J01FF (Clindamycin)	Jan/19- Jul/20	0.02	Jul-Oct/20	1.05	Oct-Dec/20	−1.04				
J01GB (Amikacin)	Jan/19- Dec/20	*0.55								
J01XA	Jan-Apr/19	2.13	Apr-Oct/19	−0.58	Oct/19- May/20	0.64	May- Aug/20	-2.15	Aug- Dec/20	1.18
Teicoplanin	Jan-Apr/19	1.78	Apr-Jul/19	−1.73	Jul/19- May/20	*0.52	May- Aug/20	-1.62	Aug- Dec/20	0.78
Vancomycin	Jan-May/19	0.78	May/19- Dec/20	*−0.09						
J01XB	Jan-May/19	−2.86	May/19- Mar/20	0.50	Mar-Jun/20	6.93	Jun-Set/20	-5.76	Sep- Dec/20	2.32
Polymyxin B	Jan-May/19	−2.88	May/19- Mar/20	0.50	Mar-Jun/20	6.50	Jun-Set/20	-5.41	Sep- Dec/20	1.97
Colistin	Jan/19- Dec/20	*0.05								
J01XD (Metronidazole)	Jan/19- Dec/20	*−0.19								
J01XX	Jan-Apr/19	1.00	Apr-Jul/19	−1.01	Jul/19-Feb/20	*0.45	Feb-Jun/20	−0.55	Jun- Dec/20	*0.69
Daptomycin	Jan/19- Dec/20	−*0.06								
Linezolid	Jan/19- Dec/20	0.04								
J02AA (Amphotericin B)	Jan/19- Dec/20	*0.05								
J02AC	Jan/19- Dec/20	0.02								
Fluconazole	Jan/19- Dec/20	−0.00								
Voriconazole	Jan/19- Mar/20	*−0.09	Mar-Dec/20	*0.25						
J02AX (Anidulafungin)	Jan/19- Dec/20	0.06								

Legend: MPC: monthly percent change

Polymyxin B, meropenem, and piperacillin/tazobactam were the most used antibiotics. When considering the yearly use, there was statistically significant growth in polymyxin B, polymyxin E, daptomycin, amikacin, and amphotericin, although these results do not consider the underlying trends for these medicines (Table 2). Regarding the anti-infectives, we observed a consumption decrease of combinations of penicillins, even though the amoxicillin and clavulanate consumption had a different trend and increased from May 2020 until the end of the analyzed period (MPC = 1.82).

**FIGURE 2:**
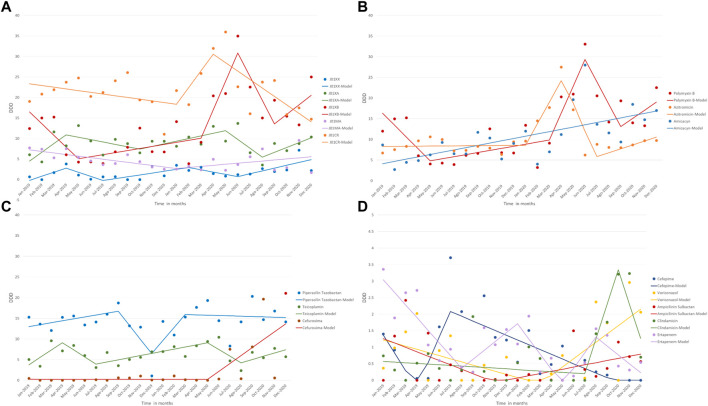
Antibiotic consumption (DDD) for **(A)** ATC groups J01XX, J01XA, J01XB, J01MA, J01CR, **(B)** Polymyxin B, Azitromicin and Amicacyn **(C)** Piperacillin Tazobactan, Teicoplamin and Cefuroxima and **(D)** Cefepime, Voriconazole, Ampicilinin Sulbactam, Clindamycin and Ertapenem from January 2019 to December 2020 (Rio de Janeiro, Brazil).

The second-generation cephalosporins (cefuroxime only used in this group) had a significant increase from May 2020 to December 2020 (MPC = 1.92) (Table and Figure 2C). On the other hand, the fluoroquinolones showed a slow and steady increasing trend from December 2019 until the end of the period analyzed (MPC = 0.26) (Table 3 and Figure 2A). In addition, azithromycin consumption increased rapidly (MPC = 5.21) from January to April 2020 (Table 3 and Figure 2B). Despite showing no change in trend, the aminoglycoside (amikacin) group consumption steadily increased over the 2 years (Table 3 and Figure 2B).

The consumption of the glycopeptide antibacterials increased from October 2019 to May 2020, falling from May to August 2020, then increasing again until December 2020. (Table 3; Figure 2A and Figure 2C). Considering the polymyxins, which include polymyxin B and colistin, there was a noticeable increase from March to June 2020 (MPC = 6.93) (Table 3 and Figure 2B). The other antibacterials (linezolid and daptomycin) had an upward trend from August to February 2020, decreasing from February to June 2020, followed by an increase in consumption from June to December 2020. (Table 3; Figure 2A and Figure 2C). Voriconazole increased from March 2020 until the end of the period analyzed. (Table 3 and Figure 2D).

Notwithstanding the general increase in antimicrobial prescription during the pandemic, there were fewer cultures with microbiological growth in 2020 compared to 2019. In 2019 2,397 cultures with microbiological growth were obtained (from 4,299 patients included) and 1,332 in 2020 (3,652 patients). MDR bacteria accounted for 39 and 27% of these cultures, respectively. (*p*-value <0.001).

Isolated non-fermenting Gram-negative bacilli increased until April 2019 (January to April 2019, MPC = 1.66^∗^). From November 2019 to February 2020, we observed a decline in the frequency of isolates. From May 2020 onwards, a decrease in isolates was observed until the end of the period analyzed. Non-fermenting Gram-negative bacilli with MDR profiles maintained an increasing baseline until April 2019 (January to April 2019; MPC 1.11). As of April 2019, there was a drop in the trend that continued until the end of the period analyzed.

Gram-negative fermenting bacilli showed an upward trend until April 2019 (January 2019 to April 2019; MPC = 2.74^∗^). In February 2020, we observed a slight increase in the trend that continued through December 2020. Gram-negative bacilli MDR fermenters had a similar tendency to global non-fermenting Gram-negative bacilli. Data are available in the supplementary material.

Gram-positive cocci showed an increase from February 2020, (MPC = 0.64) and lasted until May 2020 (MPC = 0.64). As of May, the downward trend continues until the end of the period analyzed (MPC = −0.27^∗^). Gram-positive MDR cocci have only two inflection points (January 2019 to June 2019; MPC = 0.15 and June 2019 to December 2020; MPC = −0.05^∗^).

## Discussion

This study is the first in Brazil to analyze the consumption of systemic anti-infectives in critically ill patients through the COVID-19 pandemic period. The first case of COVID-19 in the country was notified in February 2020. Brazil had 7,675,973 cases and 194,949 deaths throughout the year 2020 (Ministério da Saúde, 2021).

We found a considerable increase in anti-infective consumption in the year the pandemic began. Conversely, the microbiological profile did not correspond to consumption in terms of frequency. The peak of global antimicrobial consumption was between February and May 2020. However, there was a peak of microbiological isolates between January and April 2019. These data suggest empirical antibiotic therapy prescription without microbiological proof of infection.

This result was expected given the current guidelines recommending the use of empirical antibiotic therapy in clinical suspicion of infection (Sieswerda et al., 2021), nevertheless, they are worrying. It’s important to note that the studied hospital have been using protocols based on local epidemiology, clinical profile of patients, and availability of antimicrobials as recommended in National Health Surveillance Agency (ANVISA) guidelines (Agência Nacional de Vigilância Sanitária, 2017).

There is a great discussion in the literature about empirical antibiotic therapy prescription in COVID19 patients. On the one hand, although bacterial co-infection in viral pneumonia is relatively common (Liu et al., 2021), a systematic review including studies from China (n = 23), Singapore (n = 1), Spain (n = 3), and the USA (n = 3) indicated that bacterial co-infection in patients affected by COVID-19 was less frequent when compared to those committed by Influenza (Lansbury et al., 2020). On the other hand, Rouzé et al. (2021) states that patients affected by COVID-19 are at greater risk for respiratory tract infections when compared to patients affected by influenza or those without viral infection, assuming that COVID-19 is a risk factor for infection bacterial (Rouzé et al., 2021).

Therefore, it is unclear whether critically ill patients affected by COVID-19 are more susceptible to secondary bacterial infections (Centers for Disease Control and Prevention, 2021b). In our study, we did not observe an increase in microbiological isolates during the pandemic period, a similar result to the systematic review conducted by Lansbury et al. (2020).

Despite that, the worldwide scenario is the empirical use of antibiotics in COVID-19 patients. Studies showed that, among those COVID-19 patients that used antibiotics systematically, in about 90% of patients the prescription was empirical (Ruan et al., 2020; Yang et al., 2020; Zhou et al., 2020). This could aggravate the already serious problem of antibiotic resistance.

The WHO AWaRe classification makes it possible to visualize the antibiotics consumption that are considered the last line of treatment (World Health Organization, 2019b). We observed an increase in consumption in all groups from February 2020 onward. Although we have not found studies conducted in ICUs during the pandemic that utilized the AWaRE classification, a study involving twelve hospitals in Bangladesh drew attention to the Reserve group’s high antibiotic consumption for patients admitted in wards (Mah-E-Muneer et al., 2021).

This increase, also found in this study, is alarming. Reserve group antibiotics are intended to treat infections caused by pathogens considered critical from disseminating resistance profiles (Tacconelli et al., 2018). High priority pathogens of the WHO list, such as MDR non-fermenting Gram-negative bacilli (Tacconelli et al., 2018), can only be treated with antibiotics from the Reserve group (Doi, 2019; Karaiskos et al., 2019). The increased consumption of these antibiotics raises concerns about the therapeutic options available to treat infections in the ICU. Even more so because we did not observe an increase in the prevalence of these bacilli that would justify the increased Reserve group consumption, such as polymyxin B and ceftazidime/avibactam, indicating the empirical use of these antimicrobials.

We also found an increase in consumption of first-generation cephalosporins, fluoroquinolones, and carbapenems. These are already the most commonly used antibiotics in Brazil, according to a study that included data from ICUs, surgical clinics, and pediatrics from a teaching hospital in 2018 (Da Silva et al., 2021). Surgical sectors can consume more first-generation cephalosporins, as this class is used for surgical prophylaxis (Peel et al., 2019). In 2019, ANVISA issued a warning about the safety profile of fluoroquinolones, discouraging their use (Agência Nacional de Vigilância Sanitária, 2019).

Among the antibiotics in this group, amoxicillin/clavulanate showed an increasing trend starting May 2020 that remained until the end of the analyzed period. This association is commonly prescribed to treat community-associated pneumonia (Khilnani, et al., 2019) and, while the prevalence of bacterial co-infection in patients affected by COVID-19 is still controversial, it can not be ruled out (Contou et al., 2020; Lansbury et al., 2020). Thus, it is possible that the large number of COVID-19 patients boosted the consumption of amoxicillin/clavulanate.

Cefuroxime was the only second-generation cephalosporin in the analyzed period. The cefuroxime prescription in the studied ICU was negligible until April 2020. However, from May 2020, consumption increased significantly until the end of the period analyzed. Another study found that using second-generation cephalosporins, such as cefuroxime, did not vary significantly in the pre-pandemic period in other Brazilian hospitals (Da Silva et al., 2021). As cefuroxime, much like amoxicillin/clavulanate, is used to treat community-associated pneumonia (Khilnani, et al., 2019), it may be that the sharp upward consumption trend is explained by a large number of COVID-19 patients in the ICU.

The fluoroquinolones use trend was downward in 2019, possibly due to discouraged prescription by regulatory agencies, given its severe adverse effects (European Medicines Agency, 2018; Agência Nacional de Vigilância Sanitária, 2019; Food and Drug Administration, 2019). Nevertheless, in 2020 the consumption of fluoroquinolones increased again. It might be related to the risk of ventilator-associated pneumonia (VAP) (Wu et al., 2019) due to mechanical ventilation needed by the COVID-19 critical cases. Fluoroquinolones have a broad spectrum of action, including against *Stenotrophomonas maltophilia*, an important VAP etiologic agent (Ibn Saied et al., 2020), and COVID-19 is associated with an increased risk of VAP (Maes et al., 2021).

Usually in Brazil, the VAP diagnosis can be performed clinically without a microbiological proof (Agência Nacional de Vigilância Sanitária, 2021). Despite that, the diagnosis of VAP is controversial, especially about the necessity of cultures. European Society of Critical Care Medicine strongly recommends using cultures or molecular biology tests to diagnose VAP (Torres et al., 2017). The Infectious Diseases Society of America (IDSA), for it’s part, recommends using cultures of respiratory secretions but endorses a clinical diagnosis only (Kalil et al., 2016). Faced with the great challenge of differentiating VAP from the progressive impairment of the lung caused by COVID-19, ANVISA established specific criteria for diagnosing VAP in patients affected by COVID, among this criteria is the microbiological documentation of infection.

It would be very interesting to analyze if the patients prescribed fluoroquinolones had indeed been diagnosed with VAP. Unfortunately, the data available for this study did not allow this type of analysis.

We observed a significant increase in azithromycin consumption. However, we do not know if this sharp increase was due to medical intention to treat pneumonia promoted by atypical bacteria (Thompson et al., 2015) or in the belief that azithromycin may have some action against the SARS-COV-2 virus. Such belief was common in the Brazilian medical community at the beginning of the pandemic (Echeverría-Esnal et al., 2021). As a side note, we believe it is important to point out that WHO does not recommend using azithromycin to treat COVID-19 (World Health Organization, 2021). Moreover, the use of this antibiotic can bring critical adverse effects, especially in the cardiovascular system (Ray et al., 2012).

Daptomycin and linezolid are intended to treat infections caused by MDR Gram-positive cocci. At the end of the analyzed period (July to December 2020), there was an increase in the consumption of these antibiotics not accompanied by an increase in cases of Gram-positive MDR cocci, suggesting that many patients have received daptomycin and linezolid without microbiological proof of infection. Published data suggest that patients with viral pneumonia are susceptible to infection with *Staphylococcus aureus* MDR (Chung and Huh, 2015). Perhaps experience with previous viral pneumonia has led physicians to prescribe more antibiotics targeted to these pathogens even if microbiological evidence was lacking. A study conducted in Barcelona, Spain, also identified increased consumption of vancomycin and daptomycin, but the authors did not assess local microbiology data and assumed that consumption of these antibiotics could result from increased cases of bloodstream infection (Grau et al., 2021a).

Polymyxin B and polymyxin E are considered restricted-use antibiotics by WHO. Polymyxin showed an unprecedented increase in consumption from March 2020, nevertheless we did not observe a similar increase in cases of isolated MDR Gram-negative bacilli (fermenters and non-fermenters). Polymyxin is an antibiotic that should be reserved for infections without other therapeutic options (Zavascki et al., 2007). Its empirical use worries both microbial resistance and exhaustion of available therapeutic options (Genteluci et al., 2016) and the scope of possible adverse effects related to the drug (Vattimo et al., 2016). Even with the global increase in antibiotic consumption in the institution, we did not observe within the period analyzed an increase in MDR pathogens that could have been attributed to this increase in consumption. Furthermore, antimicrobial exposure is the main predictor for the appearance of MDR profiles (Bijnen et al., 2015). Additionally, the lack of innovation in developing new antibiotics (WHO, 2020) aggravates the scenario of microbial resistance to drugs, reinforcing efforts towards the most rational consumption possible.

Voriconazole is an azole antifungal recommended to treat invasive aspergillosis (Patterson et al., 2016), associated with critically ill patients with COVID-19 (Lai and Yu, 2021). We could not measure the empirical use because many diagnoses of invasive aspergillosis are made by detecting galactomannan in the bronchoalveolar lavage (Lai and Yu, 2021).

Our study has some limitations inherent to the study design. We analyzed the antimicrobial use trends over time, considering the COVID-19 pandemic in a single center and provided an initial overview of a health problem with some hypotheses generation. It is not the intention of this study to obtain definitive information on associations between risk factors and health outcomes.

Another limitation, all cultures collected for diagnostic purposes were considered, although some may be colonization only, and patients did not receive antibiotic therapy for the isolated pathogen. However, we were able to assess the trend in the consumption of antimicrobials for systemic use during 24 months, including nine first months of COVID-19 pandemic in the studied ICUs. Future studies are needed to measure the impact of this increase in consumption on the microbiota susceptibility profile of the intensive care units. Coronavirus 2019 disease can have a complex long-term implication for antimicrobial resistance (Rawson et al., 2020).

Joinpoint segmented regression allows identifying trend changes in time series, and many authors used this regression to analyze the consumption of antimicrobials (Peñalva et al., 2019; Guisado-Gil et al., 2020; Shafat et al., 2021). Although analyzing the data before and after the COVID-19 pandemic by comparing the years 2019 and 2020 is relevant, the trend analysis using jointpoint, considering the monthly percent change, has higher sensitivity to identify the patterns of antibiotics consumption over time. Additionally, among the possible trend analysis methods, the data-driven joinpoint analysis is a better fit since it is very challenging to determine the precise start of the pandemic. The trend analysis provides the opportunity to investigate the pattern of antibiotic consumption alongside the pandemic patterns, identifying the waves in specific antibiotics utilization. This information allows us to raise a hypothesis on how this correlates with the pandemic period, the level of scientific evidence on medicine use available at the time, and possible negative externalities due to antibiotics and microbial resistance.

## Conclusion

Overall antimicrobial consumption increased from January-2019 to December-2020 in the studied ICUs and did not correspond to the microbiological profile obtained in the same period. Despite the recommendations for empirical antibiotic therapy in clinical suspicion of infection in critically ill patients affected by COVID-19, evidence suggests that bacterial coinfection in this population is rare.

These findings are worrisome, considering that the excessive use of antimicrobials in the pandemic, especially related to surveillance and reserve drugs, can reduce available therapeutic options. Unfortunately, we do not yet know the impact of the COVID-19 pandemic on the long-term prevalence of MDR profiles. Therefore, further studies will be needed to understand the pandemic phenomenon in intensive care services.

## Data Availability

The raw data supporting the conclusions of this article will be made available by the authors, without undue reservation.
